# 胸外科围术期静脉血栓栓塞症专题前言

**DOI:** 10.3779/j.issn.1009-3419.2018.10.02

**Published:** 2018-10-20

**Authors:** 辉 李, 军 陈

**Affiliations:** 1 100020 北京，首都医科大学附属北京朝阳医院胸外科 Department of Thoracic Surgery, Beijing Chaoyang Hospital, Capital Medical University, Beijing 100020, China; 2 300052 天津，天津医科大学总医院肺部肿瘤外科 Department of Lung Cancer Surgery, Tianjin Medical University General Hospital, Tianjin 300052, China

静脉血栓栓塞症（venous thromboembolism, VTE），包括下肢深静脉血栓（deep vein thrombosis, DVT）和肺栓塞（pulmonary embolism, PE），是外科术后常见并发症之一。VTE一旦出现，会延长住院时间、增加手术死亡风险，严重影响患者术后恢复，对预后产生影响极大。一直以来，欧美以及日本等对术后VTE防治非常重视，围术期VTE预防为临床常规。国际上也有大量相关指南，例如美国胸科医师协会（American College of Chest Physicians, ACCP）静脉血栓栓塞预防及抗栓指南，美国国立综合癌症网络（National Comprehensive Cancer Network, NCCN）癌症相关性静脉血栓栓塞性疾病指南以及英国国家卫生与临床优化研究所（National Institute of Health and Clinical Excellence, NICE）静脉血栓指南等。在我国，部分外科专业如骨科、妇产科、普外科等近年也相继公布了相关共识。然而，中国胸外科对VTE的防治起步很晚，部分医师至今仍未充分认识VTE防治的必要性和重要性，而且缺乏相关临床研究和可供参考的预防共识。

鉴于此种形势，中国医疗保健国际交流促进会胸外科分会于2016年12月在国内首次成立了中国胸外科静脉血栓栓塞症研究协作组（The China National Research Collaborative Group on Venous Thromboembolism in Thoracic Surgery，以下简称研究协作组）。此后，开展了一系列VTE相关的临床研究与普及推广工作。首都医科大学附属北京朝阳医院胸外科开展了一项“肺癌术后静脉血栓栓塞症发病率的前瞻性、单中心队列研究”，发现在未经VTE预防的肺外科术后，其VTE发生率可以达到11.5%，而肺癌术后VTE发生率更高达15%。该研究近期将在欧洲胸心外科杂志（*European Journal of Cardio-Thoracic Surgery, EJCTS*）上刊出。目前，研究协作组正在开展一项前瞻性多中心临床研究进一步确认以上发现。

为提高我国胸外科VTE预防水平和规范预防方法，研究协作组于2017年成立了由来自国内胸外科、肺癌外科、呼吸危重症医学科、血管外科、药理学及护理的30余名专家组成的工作小组，共同撰写了我国首部《胸部恶性肿瘤围术期VTE预防中国专家共识》（以下简称专家共识），并将在《中国肺癌杂志》正式刊出，这是一部具有重要临床指导意义的文件。为配合专家共识的发布，编辑部还邀请了国内VTE预防开展较好的医疗中心撰写了7篇高质量的临床研究或综述文章。这组文章涵盖了胸外科术后DVT发病率和高危因素分析以及急性PE的诊断和治疗经验等多方面内容，相信对广大胸外科医生的临床工作会有较大的帮助。

但必须指出，VTE的预防仍有许多问题亟待解决，例如其他类型胸部恶性肿瘤（如食管癌、胸腺瘤等）术后VTE的发生率以及建立适合中国人群的VTE风险评估模型和药物预防方法等。这就需要我们加强合作，有计划有步骤地开展多中心前瞻性临床研究。本期专栏是一个可喜的开端，希望专家共识以及系列文献能够唤起胸外科医生对VTE预防的重视，同时了解胸外科术后VTE的发生特点，针对高危人群采取合理有效的预防措施，以降低VTE发生率和死亡率。

